# The potential role of micro-RNA 125b-5p level in predicting outcome from thrombolytic therapy in patients with acute ischemic stroke

**DOI:** 10.1007/s11239-023-02831-9

**Published:** 2023-06-08

**Authors:** Sara Mostafa, Hatem Al Masry, Mona Hussein, Rehab M. Abd Elkareem, Mohammed M. Masoud

**Affiliations:** 1 Department of Neurology, Beni-Suef University, Salah Salem Street, Beni-Suef, 62511 Egypt; 2 Department of Clinical and Chemical Pathology, Beni-Suef University, Beni-Suef, Egypt

**Keywords:** Stroke, Rt-PA, Micro-RNA 125b-5p, mRS, NIHSS

## Abstract

Several studies highlighted a significant role of specific miRNA as diagnostic and prognostic biomarkers for acute ischemic stroke. The aim of this work was to study micro-RNA 125b-5p level in patients with acute ischemic stroke in relation to stroke etiology, risk factors, severity and outcome. This case–control study was conducted on 40 patients with acute ischemic stroke eligible for receiving rt-PA and 40 age and sex matched healthy controls, Patients were submitted to neurological and radiological assessment. Functional outcome after 3 months was assessed using the modified Rankin Scale (mRS). Plasma micro-RNA 125b-5p levels were measured for both patients and control groups by quantitative real time PCR. MiRNA-125b-5p was extracted from the plasma samples then Real-time quantitative reversed transcription PCR (RT-qPCR) analysis was done. To analyze miRNA-125b-5p expression in plasma, the ∆Cq value of miRNA-125b-5p was calculated by subtracting Cq of miRNA-125b-5p from the average Cq of MiRNA RNU6B. Stroke patients had significantly higher circulating micro-RNA 125b-5p levels in comparison to healthy controls (P value = 0.01). The circulating levels of micro-RNA 125b-5p were positively correlated with stroke severity assessed by National Institutes of Health Stroke Scale (NIHSS) and infarction size. Stroke patients with poor outcome had significantly higher circulating levels of micro-RNA 125b-5p in comparison to those with good outcome (P value ≤ 0.001). The circulating levels of micro-RNA 125b-5p were significantly higher in patients who developed complications after receiving rt-PA (P value ≤ 0.001). Logistic regression model revealed that each unit increase in micro-RNA125b-5p decreased the odds of good outcome by 0.095 (95% CI 0.016–0.58, P value = 0.011). Plasma micro-RNA 125b-5p is significantly elevated is ischemic stroke patients. It is positively correlated with stroke severity and strongly associated with poor outcome and complications after thrombolytic therapy.

## Highlights

- Stroke patients had a significant upregulation in micro-RNA 125b-5p level.
- Micro-RNA 125b-5p level is positively correlated with stroke severity.
- Elevated micro-RNA 125b-5p level at stroke onset is associated with poor outcome.

## Introduction

Stroke is considered one of the medical disorders that have very high burden all over the world. According to the Global Burden of Disease (GBD), it was considered the 3rd most common cause of disability, and the 2nd most common cause of mortality after ischemic heart diseases [1] For years, the lines of stroke management were limited only to rehabilitation and secondary prevention until the approval of recombinant tissue plasminogen activator (rt-PA) as a thrombolytic therapy for stroke by FDA at 1996 [2]. It has been reported by multiple clinical trials that only 1/3 of stroke patients showed noticeable recovery after receiving rt-PA [3]. So, identification of the predictors of recovery in stroke patients who are indicated for thrombolytic therapy may be crucial for taking the proper decision regarding the management of stroke [4].

MicroRNAs (miRNAs) represent a class of noncoding RNA molecules that play an important role in regulating gene expression at the posttranscriptional level. They were reported to play an essential role in multiple biological processes such as cell proliferation and differentiation, angiogenesis, oncogenesis, inflammation and apoptosis [5]. The regulatory role of miRNAs in cerebral ischemia was addressed by several studies [6]. Some circulating miRNAs were demonstrated as potential biomarkers for diagnosis, severity, and prognosis of acute ischemic stroke (AIS) [7]. Additionally, numerous studies revealed that administration of rt-PA after AIS significantly changed the expression profile of miRNA in both the brains of animals and the blood of human [8].

A set of circulating microRNAs such as miR-125b-5p was found to be upregulated in the middle cerebral artery occlusion model. Overexpression of miR-125b-5p enhances the injury induced by oxygen and glucose deprivation (OGD) through reducing the expression of cystathionine b-synthase and subsequently the generation of hydrogen sulfide, which may be related to the antioxidant and antiapoptotic effects [9]. He et al. (2019) revealed that miR-125b-5p was strongly associated with increased stroke severity [10]. Additionally, miR-125b-5p was found to be significantly related to unfavorable 3-month outcomes in stroke patients receiving thrombolysis [11].

So, the identification of plasma levels of some miRNAs such as miR-125b-5p in patients with acute ischemic stroke may provide a better understanding of the reported variability in the outcome after thrombolytic therapy. These miRNAs can be also used as innovative targets in the treatment of acute ischemic stroke.

The aim of this work was to study micro-RNA 125b-5p level in patients with acute ischemic stroke in relation to stroke etiology, risk factors, and severity. The second objective was to clarify the potential role of micro-RNA 125b-5p level in predicting complications and outcome from thrombolytic therapy.

## Methods

### Study design

This case–control study was conducted on 40 patients diagnosed as having acute ischemic stroke (in the therapeutic window for treatment with rt-PA), and 40 age and sex matched healthy controls. The patients were recruited from Stroke Unit, Beni-Suef University Hospital, in the period from January 2020 to January 2021.

### Eligibility criteria

The study included 40 patients with acute ischemic stroke in the therapeutic window for treatment with rt-PA (first 4.5 h) and had no contraindication for receiving rt-PA (according to AHA /ASA guidelines 2018) [12]. The age range was between 18 and 85 years. The following patients were excluded from the study: patients presenting with transient ischemic attack (TIA), patients with impaired daily living before stroke onset with a pre-stroke modified Rankin Scale score (mRS) > 2, patients who underwent mechanical thrombectomy, patients with neurodegenerative diseases like Alzheimer’s disease or Parkinson’s disease, and patients with other comorbidities such as hemorrhagic blood disorders or malignancy.

### Clinical and radiological assessment

History was taken from the included stroke patients focusing on demographics and risk factors of stroke including: age, sex, hypertension (HTN), diabetes mellitus (DM), smoking, and drug abuse. Body mass index (BMI) was calculated as weight divided by height squared (kg/m^2^). The etiology of ischemic stroke in the included patients was classified based on the Trial of Org 10172 in Acute Stroke Treatment (TOAST) criteria into the following five categories: large artery atherosclerosis, small vessel disease, cardio-embolism, other determined etiology, and stroke of undetermined etiology [13].

The patients were neurologically assessed on admission before initiating intravenous infusion of rt-PA and daily for 7 days from stroke onset using National Institute of Health Stroke Scale (NIHSS) [14].

Computed tomography (CT brain) was done for all included stroke patients on admission to insure the absence of intracerebral hemorrhage or any structural lesion other than infarction. Brain CT was repeated after thrombolytic therapy (and if there was any deterioration in NIHSS) to assess the occurrence of radiological complications (edema or hemorrhagic transformation). In the follow up brain imaging, size of the infarction was measured by the pure ellipsoid model of ABC/2. It was found by Sims et al. (2009) to be the best model for a rapid and accurate clinical estimation of stroke volume [15].

Modified Rankin Scale (mRS) was done to all included patients at day 90 from stroke onset to assess the degree of disability and the dependence in the daily activities. The scale ranges from 0 to 6; from being normal without symptoms to death. Favorable and unfavorable outcomes were defined as mRS scores of 0–2 and 3–6, respectively [16].

### Laboratory assessment

Plasma micro-RNA 125b-5p levels were measured for both patients and control groups by quantitative real time PCR.

### Plasma RNA extraction

Three milliliters of EDTA-preserved venous blood samples were centrifuged at 1600×*g* at 4 °C for 20 min, and plasma was extracted and stored at − 80 °C until the assay time. MiRNA-125b-5p was extracted from plasma samples using the Qiagen miRNA Easy kit (Qiagen, Germany) according to the manufacturer's instructions to elute the RNA, including miRNA. A total of 200 µl of plasma were homogenized with 1000 µl of QIAzol lysis reagent using vortexing and pipetting and then incubated at room temperature for 20 min. The suspension was vigorously shaken before 200 µl of chloroform was added. The samples were +4 centrifuged for 15 min at 4 °C at 12,000×*g*, and the RNA in the aqueous phase was precipitated for 10 min with 400 µl isopropanol at room temperature. The RNA pellets were re-solubilized in 30 µl RNase-free water after rinsing in 1 ml ethanol (70%). The A260/280 nm ratio was used to assess the integrity and concentration of RNA using the NanoDrop ND-2000 UV spectrophotometry (Thermo Scientific, USA).

### Real-time quantitative reversed transcription PCR (RT-qPCR) analysis

Using a TaqMan^®^ MicroRNA Reverse Transcription kit, 5 µl of eluted RNA was reverse transcribed to cDNA (Applied Biosystems; Thermo Fisher Scientific, Inc.). qPCR analysis was carried out using TaqMan MicroRNA assay kits and Universal PCR Master Mix (Applied Biosystems; Thermo Fisher Scientific, Inc.) according to the manufacturer's instructions using TM Step One TM to detect the miRNAs (Applied Biosystems, Thermo Fisher Scientific). The expression of a known quantity of miRNA RNU6B, an endogenous control, was used to normalize the RT-qPCR analysis of plasma miRNA-125b-5p (Applied Biosystems; Thermo Fisher Scientific, Inc.). To analyze miRNA-125b-5p expression in plasma, the ∆Cq value of miRNA-125b-5p was calculated by subtracting Cq of miRNA-125b-5p from the average Cq of MiRNA RNU6B.

### Sampling

The sample size calculation was done using G*Power version 3.1.9.2 Software. We calculated the sample size based on the results of a pilot study we performed before starting our study. The effect size = 0.568, the probability of type I error (α) was 5%, critical t = 1.665, df = 78, and noncentrality parameter δ = 2.541. A total sample size of 40 patients in each group was required to reach a statistical power (1 − β) 80%.

### Statistical analysis

IBM SPSS Version 25 was used to analyze the data. Categorical variables were expressed as numbers and percentages and normally distributed quantitative variables were expressed as mean and standard deviation (SD). Chi-squared test was used for comparison between groups in categorical variables and the Independent sample t-test was used to compare between groups in normally distributed quantitative variables. ANOVA test was used for comparison between three groups in quantitative variables. Correlations between miR-125b-5p level and NIHSS and infarction size were done using Pearson correlation test. A binary logistic regression model was done to identify if miR-125b-5p level can be a predictor of complications and outcome from thrombolytic therapy after being adjusted for their potential mutual confounding effect. P value ≤ 0.05 was considered statistically significant. All tests were two-tailed.

## Results

### Demographics, clinical, radiological and laboratory characteristics of the included stroke patients

The mean age of rt-PA treated patients was 61.25 ± 8.28 years, while the mean age of controls was 60.44 ± 7.74 years. Regarding sex, 52.5% (n = 21) of rt-PA treated patients were males and 47.55% (n = 19) were females, while, 42.5% (n = 17) of controls were males and 57.5% (n = 23) were females. There were no statistically significant differences between patients and controls regarding age or sex (P value = 0.356, 0.73 respectively).

Data regarding, risk factors of stroke, TOAST classification, mean duration of stroke, mean door to needle time, NIHSS before and 7 days after thrombolysis, and infarction size at day 7, were illustrated in Table 1. Only 5 (12.5%) patients developed complications from rt-PA (2 patient developed ICH and 3 patients developed edema). Regarding outcome as assessed by mRS, 13 (32.5%) patients had good outcome, whereas 27 (67.5%) patients had poor outcome (Table 1).

**Table 1:** Demographics, clinical and radiological characteristics of the included stroke patients

	Patients (n = 40)	Controls (n = 40)	P value
Age [Mean (SD)]	61.25 (8.28)	60.44 (7.74)	0.791
Sex			0.73
Males [N (%)]	21 (52.5%)	17 (42.5%)	
Females [N (%)]	19 (47.55%)	23 (57.5%)	
Risk factors of stroke			
BMI [mean (SD)]	29.19 (5.98)		
DM [N (%)]	15 (37.5%)		
HTN [N (%)]	22 (55%)		
AF [N (%)]	17 (42.5%)		
Smoking [N (%)]	17 (42.5%)		
Drug abuse [N (%)]	1 (2.5%)		
TOAST classification			
Large artery stroke [N (%)]	13 (32.5%)		
Cardio-embolic stroke [N (%)]	17 (42.5%)		
Small artery stroke [N (%)]	10 (25%)		
Duration of stroke (in min) [mean (SD)]	160.5 (47.86)		
Door to needle time (in min) [mean (SD)]	42.12 (19.07)		
NIHSS before thrombolysis [mean (SD)]	14.97 (3.66)		
NIHSS after 7 days [mean (SD)]	9.45 (8.01)		
Infarction size at day 7 [mean (SD)]	12.6 (9.28)		
Complications of rt-PA			
Had [N (%)]	5 (12.5%)		
Didn’t have [N (%)]	35 (87.5%)		
mRS after 3 months			
Good outcome [N (%)]	13 (32.5%)		
Poor outcome [N (%)]	27 (67.5%)		

*AF* atrial fibrillation, *BMI* body mass index, *DM* diabetes mellitus, *DTN* door to needle**,** *HTN* hypertension, *mRS* modified Rankin scale, *NIHSS* National Institute of Health Stroke Scale, *rt-PA* recombinant tissue plasminogen activator, *TOSAT* Trial of Org 10172 in Acute Stroke Treatment

P value > 0.05 is considered non-significant

**Fig. 1:**
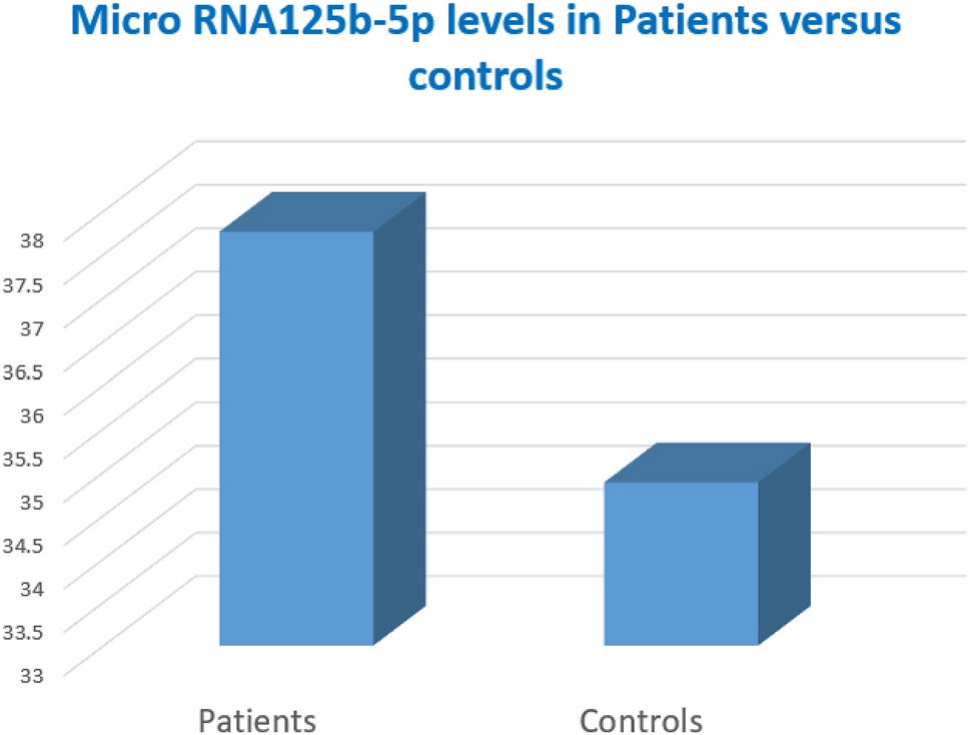
Micro RNA125b-5p levels in the included stroke patients and controls

The mean value for micro-RNA 125b-5p level in rt-PA treated patients was 37.76 ± 1.73, while in controls was 34.88 ± 2.65 (FC). There was a statistically significant difference between patients and controls (P value = 0.012) (Fig. 1).

### Micro-RNA125b-5p level in the included stroke patients in relation to risk factors and etiology of stroke

There was no effect of either DM, HTN, AF, smoking, or drug abuse on micro-RNA125b-5p level in patients with acute ischemic stroke (P value = 0.311, 0.925, 0.59, 0.146, 0.197 respectively). There was no statistically significant difference between patients with large artery stroke, those with cardio-embolic stroke, and those with small artery stroke regarding miRNA125b-5p levels (P value = 0.61) (Table 2).

**Table 2:** micro-RNA125b-5p level in the included stroke patients in relation to risk factors and etiology of stroke

Risk factors and etiology of stroke	Micro-RNA125b-5p(FC) [mean (SD)]
Risk factors of stroke	
DM	
With (n = 15)	37.37 (2.06)
Without (n = 25)	38 (1.5)
P value	0.311
HTN	
With (n = 22)	37.79 (1.74)
Without (n = 17)	37.73 (1.78)
P value	0.925
AF	
With (n = 17)	37.59 (1.81)
Without (n = 23)	37.89 (1.70)
P value	0.59
Smoking	
Smokers (n = 17)	38.23 (1.53)
Non-smokers (n = 23)	37.42 (1.83)
P value	0.146
Drug abuse	
Drug abusers (n = 1)	40
Not drug abusers (n = 39)	37.7 (1.72)
P value	0.197
TOAST classification	
Large artery stroke (n = 13)	38.17 (1.68)
Cardio-embolic stroke (n = 17)	37.59 (1.82)
Small artery stroke (n = 10)	37.55 (1.77)
P value	0.61

*AF* atrial fibrillation, *DM* diabetes mellitus, *HTN* hypertension, *N* number, *RNA* ribonucleic acid, *TOSAT* Trial of Org 10172 in Acute Stroke Treatment

P value > 0.05 is considered non-significant

### The impact of micro-RNA125b-5p level on stroke severity, complications and outcome from thrombolytic therapy

There were statistically significant positive correlations between micro-RNA 125b-5p circulating level and NIHSS before and 7 days after thrombolysis, and infarction size at day 7 (P value ≤ 0.001 in three correlations) (Table 3).

**Table 3:** Correlation between micro-RNA125b-5p level and NIHSS and infarction size before and after thrombolysis

	Micro-RNA125b-5p(FC)	
	(r) Coef.	P value
NIHSS before thrombolysis	0.544	≤ 0.001*
NIHSS at day 7	0.854	≤ 0.001*
Infarction size at day 7	0.887	≤ 0.001*

*NIHSS* National Institute of Health Stroke Scale, *RNA* ribonucleic acid

(r): Pearson coefficient, *P value ≤ 0.05 is considered significant

Patients with complication from rt-PA had significantly higher circulating micro-RNA 125b-5p level than those without (P value ≤ 0.001). Also, patients with poor outcome after thrombolysis had significantly higher circulating micro-RNA 125b-5p level than those without (P value ≤ 0.001) (Table 4).

**Table 4:** Impact of micro-RNA 125b-5p level on the occurrence of complications and on the outcome from thrombolytic therapy

	Micro-RNA 125b -5p(FC) [mean (SD)]
rt-PA complications	
Had [n = 5]	39.69 (0.43)
Didn’t have [n = 35]	37.49 (1.68)
P value	≤ 0.001*
mRS after 3 months	
Good outcome [n = 13]	35.93 (0.84)
Poor outcome [n = 27]	38.64 (1.31)
P value	≤ 0.001*

*mRS* modified Rankin Scale, *RNA* ribonucleic acid, *rt-PA* recombinant tissue plasminogen activator,

*P value ≤ 0.05 is considered significant

The binary logistic regression revealed that micro-RNA125b-5p level was not a predictor of complications from thrombolytic therapy (OR = 4.255, P value = 0.06). But regarding outcome, each unit increase in micro-RNA125b-5p decreased the odds of good outcome by 0.095 (CI 0.016–0.58, P value = 0.011). (Table 5).

**Table 5:** Logistic regression model to determine if micro-RNA125b-5p level can be a predictor of complications and outcome from thrombolytic therapy

Dependent variables	Independent variables	B	Chi square	P value	Odds ratio	95% CI	
						Lower	Upper
rt-PA complications	micro-RNA125b-5p(FC)	1.448	3.538	0.060	4.255	0.941	19.241
	Constant	− 58.323	3.661	0.056	0.000		
mRS after 3 months	Micro-RNA125b-5p(FC)	− 2.352	6.501	0.011*	0.095	0.016	0.58
	Constant	86.133	6.539	0.011	2.55E+37		

*CI* confidence interval, *RNA* ribonucleic acid

*P value ≤ 0.05 is considered significant

## Discussion

Several studies have highlighted the role of some miRNA as potential diagnostic and prognostic biomarkers for acute ischemic stroke. Recently, much concern was directed towards the potential use of certain miRNAs as innovative targets in the treatment of stroke [17]. The aim of this work was to study micro-RNA 125b-5p level in patients with acute ischemic stroke in relation to stroke etiology, risk factors, severity and outcome.

Our results revealed that stroke patients had significantly higher circulating micro-RNA 125b-5p levels in comparison to healthy controls.

Sepramaniam et al. (2014) first clarified the differences in expression of miRNAs between patients with acute stroke and those with old stroke. They found that miR‐125b levels were significantly upregulated in patients with acute stroke compared to those with old stroke [18]. A subsequent study conducted by Tiedt et al. (2017), which enrolled a larger number of patients, revealed that plasma miR‐125b-5p levels rose rapidly after developing acute ischemic stroke and then restored two days later [19].

Tiedt et al. (2017) addressed the combination of miR-125a-5p, miR-125b-5p and miR-143-3p as significant diagnostic biomarkers for AIS with remarkable sensitivity (85.6%), specificity (76.3%) and AUC value (0.90). They were also elevated in patients with AIS when compared with patients with TIA. Their findings suggest platelets as a major source of elevated miR-125a-5p, miR-125b-5p, and miR-143-3p. Whether this relates to thrombus formation, platelet aggregation or not, was still unclear [19].

Neuronal cell apoptosis is considered the main pathology in AIS [20]. Current evidence has suggested a crucial role of certain miRNAs in regulating neuronal death in AIS [21]. Also, some miRNAs such as miR-125b-5p were implicated in other pathways related to cerebral ischemia, such as inhibition of angiogenesis [22]. Besides, miR-125a-5p has been reported to play an important role in the differentiation of inflammatory cells [23].

Zampetaki et al. (2012) suggested platelets as an important source of circulating miRNAs 125 b-5p [24]. Kaudewitz et al. (2016) tried to determine the extent to which platelets may contribute to increase the levels of miR-143-3p, miR-125a-5p, and miR-125b-5p. They isolated platelets from platelet-rich plasma (PRP) and spiked them back into platelet-poor plasma at increasing concentrations. The found that the levels of all 3 miRNAs showed significant elevation with increasing concentrations of platelets [25].

In the present study, the circulating levels of Micro RNA 125b-5p were positively correlated with stroke severity assessed by NIHSS and infarction size.

Similar to our findings, He et al. (2019) conducted a prospective cohort study on 94 patients with AIS. They measured the plasma levels of miR-125b-5p, 24 h after thrombolytic therapy with or without endovascular treatment. Stroke severity was assessed based on NIHSS score and infarct size. They found that miR-125b-5p levels were positively correlated with NIHSS scores and infarct volumes. Also, miR-125b-5p levels were significantly higher in patients with moderate-to-severe stroke in comparison to those with mild stroke [11].

Liu et al. (2019) found that the circulating level of miR-128, 125b-5p, 124b, were positively correlated with infarction volume, NIHSS score at 7 days and mRS score at 3 months. They explained their findings by highlighting the association between the circulating level of miR-128, 125b-5p, 124b, and both neuro-inflammation and neuronal cell death [26].

In contrast to our findings, Tiedt et al. (2017) found that the expression levels of miR-125a-5p, miR-125b-5p, and miR-143-3p were independent of infarct volume. They emphasized the potential role of these biomarkers as adjuncts in the diagnosis of AIS regardless of stroke severity [19].

In the present study, the circulating levels of micro-RNA 125b-5p were significantly higher in patients who developed complications (ICH or reperfusion injury) after receiving rt-PA. Nevertheless, the logistic regression model revealed that micro-RNA125b-5p level was not a predictor of complications from thrombolytic therapy. He et al. (2019a) found that there were no associations between the plasma levels of miR-125b-5p and post thrombolysis ICH [10].

Regarding outcome from thrombolysis, our results revealed that patients with poor outcome had significantly higher circulating levels of micro-RNA 125b-5p in comparison to those with good outcome. Additionally, logistic regression model revealed that each unit increase in micro-RNA125b-5p decreased the odds of good outcome by 0.095.

In accordance with our findings, Rainer et al. (2016) indicated that miRNA 125b-5p could be used as a prognostic biomarker in AIS patients receiving thrombolysis [8]. Similar findings were obtained by He et al. (2019a, b) who found that miR-125b-5p level was an independent predictive marker for unfavorable outcome (mRS > 2) after thrombolysis, with AUC value, sensitivity and specificity of 0.735, 86.36% and 55.36% [10, 11]. Likewise, Liu et al. (2019) found that the circulating level of 125b-5p was positively correlated mRS score at 3 months after an ischemic stroke [26].

Following cerebral ischemic event, changes in the miRNA 125b-5p transcriptome indicated the role of miRNAs 125b-5p in some ischemic pathological events such as excitotoxicity which might affect the disease outcome [27]. The analysis of stroke patients shows that miR-125b-5p manifests its maximum expression level within the acute phase of stroke [18]. The overexpression of miR-125b-5p which occurs after ischemic stroke can upregulate NMDA receptors (NR2A), which promote cell death and exacerbate post-stroke excitotoxicity [28].

In the present study, there was no effect of either DM, HTN, or smoking on micro-RNA 125b-5p level. Similar to our findings, Tiedt et al. (2017) found that micro-RNA 125 b -5p level in stoke patients was not affected by diabetes, HTN or smoking [19].

In our study, there was no statistically significant difference between patients with large artery stroke, those with cardio-embolic stroke, and those with small artery stroke regarding miRNA125b-5p levels.

In accordance with our findings, Tiedt et al. (2017) found that miR-125b-5p was similar across etiologic subgroups of stroke patients; large vessel stroke, cardio embolic stroke, and stroke of undetermined pathogenesis [19]. On the other hand, Gui et al. (2018) demonstrated that cardio embolic stroke patients exhibited significantly higher circulating miR‐125b-5plevels than both atherosclerotic ischemic stroke and healthy control [29].

Our work has some limitations. Firstly, the small sample size. Secondly, we measured micro-RNA 125 level only before thrombolysis and not after. So, the effect of thrombolysis on micro-RNA 125 expression profile was not clarified in our study. Thirdly, we didn’t investigate the role of micro-RNA 125b-5p as a predictor of functional outcome after thrombectomy.

## Conclusion

Stroke patients had significantly higher circulating micro-RNA 125b-5p levels in comparison to healthy controls. The circulating levels of micro-RNA 125b-5p were positively correlated with stroke severity, and was significantly higher in patients who developed complications (ICH or reperfusion injury) after receiving rt-PA and in those with poor outcome.

## Data Availability

Authors report that the datasets used and/or analyzed during the current study are available from the corresponding author on reasonable request.
